# No evidence that facial width-to-height ratio (fWHR) is associated with women's sexual desire

**DOI:** 10.1371/journal.pone.0200308

**Published:** 2018-07-18

**Authors:** Weiqing Zhang, Amanda C. Hahn, Ziyi Cai, Anthony J. Lee, Iris J. Holzleitner, Lisa M. DeBruine, Benedict C. Jones

**Affiliations:** 1 Institute of Neuroscience & Psychology, University of Glasgow, Glasgow, United Kingdom; 2 Department of Psychology, Humboldt State University, Arcata, United States of America; Macquarie University, AUSTRALIA

## Abstract

Facial width-to-height ratio (fWHR) has been linked to many different behavioral tendencies. However, not all of these correlations have replicated well across samples. Arnocky et al. (in press, Archives of Sexual Behavior) recently reported that sexual desire was correlated with fWHR. The current study aimed to test this relationship in a large sample of women. fWHR was measured from face images of 754 women. Each woman completed the Sexual Desire Inventory, which measures total, dyadic, and solitary sexual desire. Analyses revealed no significant correlations between fWHR and any of our measures of sexual desire. These null results do not support the hypothesis that fWHR is related to women’s sexual desire. Additionally, we found no evidence that women’s face-shape sexual dimorphism was related to their sociosexual orientation.

## Introduction

Many recent studies have reported correlations between facial width-to-height ratio (face width scaled for face height, fWHR) and behavioral tendencies (for recent reviews see [1–4]). For example, several studies have reported that people with higher fWHR are more aggressive (see [2] and [3] for meta-analytic reviews). Although the mechanism through which correlations between fWHR and behavioral tendencies emerge is not known, some researchers have hypothesized that they occur because testosterone influences both fWHR and behavioral tendencies [5]. This explanation is somewhat contentious, however [6]. For example, neither circulating testosterone [1] nor prenatal testosterone exposure [7] reliably predict fWHR and evidence for an association between circumpubertal testosterone and fWHR is mixed [5,6].

Some recent large-scale studies of putative relationships between fWHR and behavioral tendencies have not replicated the significant correlations reported in previous studies [4]. Moreover, some previously reported findings for fWHR, such as an association between circulating testosterone and fWHR, have not been supported by subsequent meta-analyses [1]. Null results like these arguably raise concerns about the replicability of fWHR findings [1,4].

Arnocky et al. [8] recently reported that people with higher fWHR (as measured from face photographs) reported greater sexual desire (Study 1: N = 145; Study 2: N = 314). The strength of this correlation did not differ significantly between men and women, although the correlation was significant in men, but not women, when the sexes were analyzed separately. Consequently, Arnocky et al. [8] concluded that fWHR is a correlate of sexual desire in both men and women and a potentially important cue of individual differences in sexual desire. No other study has tested this possible relationship between fWHR and sexual desire. This is potentially important, given concerns about the replicability of fWHR findings. The stated rationale for investigating the relationship between fWHR and sexual desire was that sexual desire and fWHR are both influenced by testosterone levels [8]. Given the association between testosterone and sexual desire may not be robust (see earlier discussion), the theoretical basis for a correlation between fWHR and sexual desire is also arguably rather weak.

In light of the above, we investigated the putative correlation between fWHR and sexual desire (assessed using Spector et al’s [9] Sexual Desire Inventory, SDI-2) in a large sample of women (N = 754). Arnocky et al. [8] also predicted (but did not find) that sexual desire would be correlated with sexually dimorphic face shape in women. Consequently, we also tested whether sexual desire was correlated with two different measures of sexual dimorphism of face shape.

In addition, Campbell et al. [10] found that women with more masculine face shapes reported being more open to short-term sexual relationships. Because of this finding, we also investigated possible relationships between women’s face shapes and their scores on the revised Sociosexual Orientation Inventory (SOI-R [11]).

## Methods

### Participants

Seven hundred and fifty-four young adult women took part in the study (mean age = 21.52 years, SD = 3.18 years), which was part of a larger project on hormones and mating psychology [12–14]. All of the women who participated in the study were students at the University of Glasgow. This work was approved by University of Glasgow's Psychology Ethics Committee

### Face photography

We used a Nikon D300S digital camera to take a full-face digital photograph of each woman in a small windowless room, against a constant background, and under standardized diffuse lighting conditions. Participants posed with neutral expressions. Camera settings and camera-to-head distance were held constant.

### Facial metrics

fWHR (M = 2.15, SD = 0.14) was measured from each face photograph using an identical procedure to the one reported in Lefevre et al. [15]. Face height was the distance between the upper lip and the highest point of the eyelids. Face width was the maximum distance between the left and right facial boundary (i.e., bizygomatic width).

Sexual dimorphism of face shape was objectively measured from each face photograph using two methods: a discriminant analysis method (see [16] and [17] for methods) and vector analysis method (see [18] and [19] for methods). These methods use shape components derived from principal component analyses of facial landmarks to measure the probability of the face being classified as male (for the discriminant analysis method) or to locate the face on a female-male continuum (for the vector analysis method). Code for calculating discriminant and vector scores is publicly available at https://osf.io/98qf4/. Higher discriminant scores (M = -0.90, SD = 0.93, min = -4.52, max = 2.14) or higher vector scores (M = 0.19, SD = 0.41, min = -1.32, max = 1.48) indicate more masculine face shapes. Discriminant scores and vector scores were positively correlated (rho = .56, N = 754, p < .001). fWHR was not significantly correlated with either discriminant scores (rho = -.05, N = 754, p = .18) or vector scores (rho = -.03, N = 754, p = .35).

**Fig 1:**
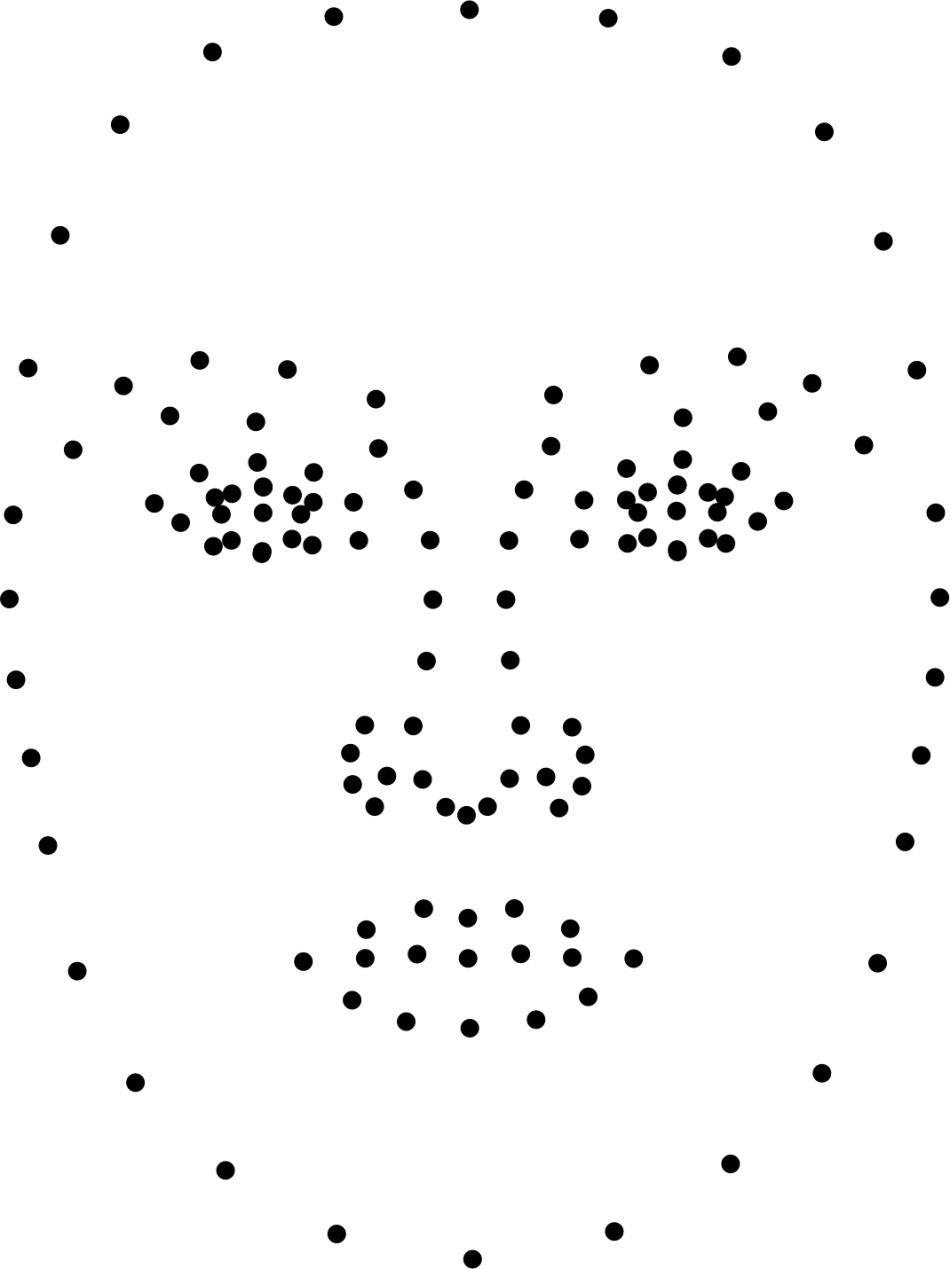
The points used for all facial metrics in our study. Points used to measure fWHR are shown in red. All points, both black and red, were used to measure vector and discriminant scores.

The points used for all facial metrics are shown in Fig 1.

### Sexual Desire Inventory (SDI-2)

The Sexual Desire Inventory (SDI-2) is a 14-item questionnaire that assesses general sexual desire [9]. An example question is “When you are in romantic situations (such as a candle lit dinner, a walk on the beach, etc.), how strong is your sexual desire?” Participants responded using a 1 (no desire) to 9 (strong desire) scale.

As well as providing a score for total sexual desire (M = 47.57, SD = 13.78), the SDI-2 also provides separate scores for desire for sexual activity with another person (dyadic sexual desire, M = 38.39, SD = 10.15) and desire for sexual activity by oneself (solitary sexual desire, M = 9.07, SD = 6.26). Thirty women chose not to complete the solitary sexual desire subscale, 28 women chose not to complete the dyadic sexual desire subscale, and 710 women completed both subscales (i.e., total sexual desire scores were available for 710 women). Higher scores on each subscale indicate greater sexual desire.

### Sociosexual Orientation Inventory (SOI-R)

Participants completed the 5-point response scale version of the SOI-R [11]. The questionnaire consists of 9 items, each of which is answered using a 1 to 5 scale. The SOI-R has three components (behavior, attitudes, and desires). Scores for each component are calculated by summing the individual scores for the 3 relevant items. Higher scores indicate more unrestricted sociosexuality (i.e., greater openness to short-term relationships). The SOI-R behavior component consists of 3 items (e.g., “With how many different partners have you had sex within the past 12 months?”), for which 1 on the response scale corresponds to “0 sexual partners” and 5 corresponds to “8 or more sexual partners” (M = 6.32, SD = 2.74). The SOI-R attitudes component consists of 3 items (e.g., “Sex without love is OK”), for which 1 on the response scale corresponds to “totally disagree” and 5 corresponds to “totally agree” (M = 9.68, SD = 3.42). The SOI-R desires component consists of 3 items (e.g., “In everyday life, how often do you have spontaneous fantasies about having sex with someone you have just met?”), for which 1 on the response scale corresponds to “never” and 5 corresponds to “nearly every day” (M = 8.00, SD = 2.94). Twenty-four women chose not to complete the behavior subscale, 10 women chose not to complete the attitude subscale, and 14 women chose not to complete the desire subscale. Higher scores on each subscale indicate greater openness to uncommitted sexual relationships.

### Results

Not all of our variables were normally distributed. Consequently, we tested for significant correlations between each of our three face shape measures (fWHR, vector score, discriminant score) and questionnaire scores using Spearman's rank correlation coefficient (i.e., Spearman’s rho). Data are publicly available at https://osf.io/pw3tj/. SPSS v21 was used to carry out analyses. None of our face shape measures were significantly correlated with total sexual desire, dyadic sexual desire, or solitary sexual desire (see Table 1). Similarly, none of our face shape measures were significantly correlated with sociosexual attitudes, desires, or behaviors (see Table 2).

**Table 1:** Correlations between our three face shape measures (fWHR, vector score, and discriminant score) and total sexual desire, dyadic sexual desire, and solitary sexual desire.

	total sexual desire			dyadic sexual desire			solitary sexual desire		
	rho	N	p	rho	N	p	rho	N	p
fWHR	-.01	710	.85	-.02	726	.63	.001	724	.97
vector score	-.02	710	.68	.003	726	.94	-.003	724	.94
discriminant score	-.01	710	.82	-.02	726	.59	.04	724	.31

**Table 2:** Correlations between our three face shape measures (fWHR, vector score, and discriminant score) and sociosexual attitudes, desires, or behaviors.

	sociosexual attitudes			sociosexual desires			sociosexual behaviors		
	rho	N	p	rho	N	p	rho	N	p
fWHR	-.01	746	.48	-.03	740	.48	.03	730	.50
vector score	-.01	746	.84	-.06	740	.10	.03	730	.40
discriminant score	.00	746	.97	-.04	740	.31	.00	730	.92

Repeating the analyses of fWHR described above using fWHR measured using the same method as Arnocky et al. [8] did not alter the pattern of null results (all absolute r < .04, all p>.31). The two measures of fWHR were positively and significantly correlated (rho = .84, N = 754, p < .001).

## Discussion

Arnocky et al. [8] recently reported a positive relationship between fWHR and sexual desire. Because the strength of this correlation was not modulated by participant sex, Arnocky et al. [8] concluded that fWHR was potentially a valid cue of sexual desire in both men and women. Our analyses of fWHR and sexual desire in a large sample of women found no evidence that women’s sexual desire was related to their fWHR. Neither total, dyadic, nor solitary sexual desire was significantly correlated with fWHR. Like Arnocky et al. [8], we saw no evidence that face-shape sexual dimorphism was significantly correlated with sexual desire or sociosexual orientation.

Our findings are the most recent example of findings for behavioral tendencies and fWHR not replicating well in larger samples (see also, e.g., [3] and [4]). These non-replications raise the important question of why fWHR-behavior correlations appear so fragile. One possibility is that measurement of fWHR from 2D images is particularly prone to measurement error. For example, even subtle changes in head position, expression, and camera settings can have large effects on fWHR measurements [20,21]. This measurement error, in combination with publication bias, may increase the rate at which false positives are present in the published literature on correlations between fWHR and behavioral tendencies. We also used a different measure of sexual desire to the one used in Arnocky et al. It is possible that subtle differences in the aspects of sexual desire measured by these instruments also contribute to differences in results across studies.

The null results we report for sexual desire and women’s fWHR do not necessarily imply that the correlation between sexual desire and fWHR reported by Arnocky et al. [8] was a false positive. Although Arnocky et al. [8] found no evidence that the strength of the correlation between fWHR and sexual desire differed significantly between men and women, the correlation was not significant when women’s data were analyzed separately from men’s data. Thus, the relationship Arnocky et al. [8] observed for sexual desire and fWHR may have been driven primarily by men’s responses and face shapes.

In conclusion, despite having a very large sample size, we did not replicate Arnocky et al’s [8] finding that higher fWHR was associated with greater sexual desire. We suggest that our null results highlight the importance of replication in large samples for establishing the reliability of putative relationships between fWHR and behavioral tendencies.
